# Autogenous Tooth Transplantation of Canines—A Prospective Clinical Study on the Influence of Adjunctive Antibiosis and Patient-Related Risk Factors During Initial Healing

**DOI:** 10.3390/jcm14030821

**Published:** 2025-01-26

**Authors:** Sebastian Meinzer, Dirk Nolte, Karin Christine Huth

**Affiliations:** 1 Practice Clinic for Oral and Maxillofacial Surgery, Sauerbruchstraße 48, 81377 Munich, Germany; dirk.nolte@mkg-muc.com; 2 Department of Conservative Dentistry and Periodontology, LMU University Hospital, LMU Munich, Goethestraße 70, 80336 Munich, Germany; karin.huth@med.uni-muenchen.de; 3 TUM School of Medicine and Health, University Hospital Rechts der Isar, Technische Universität München (TUM), Ismaninger Straße 22, 81675 Munich, Germany; 4 Department of Oral and Maxillofacial Surgery, LMU University Hospital, LMU Munich, Lindwurmstraße 2a, 80337 Munich, Germany

**Keywords:** autogenous tooth transplantation, canines, prospective study, initial healing, adjunctive antibiosis, risk factors

## Abstract

**Objectives**: This prospective clinical study investigated the efficacy of adjunctive antibiotic therapy (doxycycline) and the patient’s risk factors during initial healing of autogenous canine tooth transplantations. **Methods**: Sixty-seven patients (ranging from 11 to 37 years of age) treated with tooth transplantations were allocated to three parallel groups based on the tooth’s intraoperative extraoral storage time (EST 0–3, 4–6, and 7–15 min) receiving different antibiotic regimens: (1) no antibiotics; (2) intraoperative intravenous (i.v.) single-shot antibiotics; and (3) intraoperative i.v. single-shot plus postoperative oral antibiotics for five days. Initial healing was rated according to pain intensity and clinical signs of pathology over a 21-day period. The influence of the following parameters was investigated using RStudio (linear regression and partial eta squared statistics): group, sex, age, nicotine abuse, tooth apex condition, preoperative ankylosis, displacement severity, jaw location, the number of simultaneous transplantations and other interventions, preoperative orthodontic extrusion, EST, and intraoperative complications. **Results**: No significant influence for sex (43 females, 24 males), tooth apex condition (19 open, 48 closed), displacement severity, jaw location (51 upper, 16 lower jaw), EST (mean 4.99 min), intraoperative complications (*n* = 13), or antibiotic regimen on pathology signs or pain intensity were found. Six patients reported medication side effects. Preoperative ankylosis (*n* = 15) and unsuccessful orthodontic extrusion (*n* = 16) increased postoperative pain (*p* = 0.020, ηP^2^ = 0.08; *p* = 0.035, ηP^2^ = 0.07). Multiple transplants (*n* = 14) and interventions in multiple regions (*n* = 27) affected pain and pathology (*p* = 0.002, ηP^2^ = 0.14; *p* = 0.001, ηP^2^ = 0.17). Increased age and nicotine abuse (*n* = 6) were associated with increased pathology signs (*p* = 0.024, ηP^2^ = 0.08; *p* = 0.029, ηP^2^ = 0.07). **Conclusions**: The results suggest that personalized rather than routine antibiotic therapy might be sufficient for initial healing in canine tooth transplantation. Deteriorating factors include preoperative ankylosis, orthodontic extrusion, an increased number of surgical sites, age, and nicotine abuse. **Clinical Significance**: Routine antibiotic prevention regimes may not be mandatory for initial healing in autogenous tooth transplantation, but a nuanced antibiotic strategy tailored to each patient’s specific risk factors, which is in line with the principle of antibiotic stewardship, is needed.

## 1. Introduction

Tooth impaction and retention are common eruption disorders that affect approximately 2% of the global population [1]. These conditions often arise due to the unique, long S-shaped eruption path of certain teeth, particularly permanent canines [2]. Impaction refers to a deviation from the normal eruption direction [3], while retention describes a failure of the tooth to erupt within the expected timeframe [4]. Various factors contribute to these disorders, including space constraints, mechanical retention (e.g., root hooks or severe impaction), tumors, and malformations associated with syndromes such as cleidocranial dysostosis or Down syndrome [5]. These conditions can lead to significant impairments in dentition and occlusion, such as the resorption of adjacent teeth, functional disturbances in chewing, and even respiratory complications [6].

The management of impacted teeth typically prioritizes orthodontic extrusion, which might be supported by surgical exposure to guide the impacted tooth into its correct position [7]. However, when orthodontic treatment fails or is not feasible or unsuccessful, surgical alternatives are explored, including the extraction of the impacted tooth or autogenous tooth transplantation [8].

Autogenous tooth transplantation involves the removal of a tooth or tooth germ from its original site and its placement into a suitable recipient site within the same patient [9]. This technique offers a promising solution to integrate the tooth into the dental arch in the correct position. Indications for autotransplantation include severe displacement with the resorption of adjacent teeth, mechanical retention, delayed physiological eruption by more than two years, or unsuccessful attempts to integrate the tooth through orthodontic methods, such as exposure and bracketing [10,11,12]. Recent studies have demonstrated high success rates for this approach. For example, a retrospective study reported a 93.4% survival rate for transplanted canines (378 teeth) over an average follow-up period of five years [13], and a meta-analysis further confirmed the favorable outcomes of this technique on a larger scale [14].

Due to the invasive nature of autotransplantation and the associated risk of complications, perioperative antibiotics are often used as a precautionary measure. However, there is no clear evidence-based guideline for their use, and the existing literature shows mixed results [15,16]. This uncertainty is particularly relevant for adolescents, the primary patient group for autotransplantation, who may be more susceptible to antibiotic-related side effects due to their developing immune and digestive systems [17]. Additionally, the routine use of antibiotics raises concerns about the potential development of antibiotic resistance [18,19]. Tetracyclines, such as doxycycline, are often chosen in dental traumatology for their anti-resorptive properties [20]. Nevertheless, the decision to use antibiotics typically rests with the clinician and requires a careful risk–benefit analysis. A meta-analysis examining antibiotic use in cases of tooth replantation suggests that healing outcomes may not be significantly influenced, questioning their general necessity [21].

This prospective clinical trial aims to evaluate the effect of adjunctive antibiotic therapy along with patient-specific risk factors on initial healing and the management of complications following autogenous tooth transplantation. The rationale for this investigation is to minimize potentially unnecessary medication use and its associated risks. Our hypothesis was that standardized perioperative antibiotic therapy is not mandatory for the initial healing of canine transplants and that patient-specific risk factors play a more significant role.

## 2. Materials and Methods

### 2.1. Trial Design and Participants

Within this prospective clinical trial, participants were enrolled, and interventions as well as data analysis were performed at the Praxisclinic for Oral and Maxillofacial Surgery (Sauerbruchstraße 48, 81377 Munich, Germany) in collaboration with the Department of Conservative Dentistry and Periodontology, University Hospital, LMU Munich (Goethestraße 70, 80336 Munich, Germany) between 2021 and 2023. The study was approved by the local ethics committee (LMU Munich; Project number: 20-0903), registered by the German Clinical Trials Register (ID: DRKS00034011), and its report was based on CONSORT [22]. The cohort comprised 67 patients aged between 11 and 37 years.

#### 2.1.1. Inclusion Criteria

The inclusion criterion included an indication of the autogenous transplantation of a displaced canine. This is given in cases of the definite retention of the tooth, often following unsuccessful attempts at orthodontic alignment, with most referrals coming from orthodontists [23,24]. Further inclusion criteria were informed written patient consent and parental/guardian consent for minors, a minimum age of 11 years considering the indication range of doxycycline [25], as well as acceptable oral hygiene (gingiva, plaque, and calculus index ≤ 1).

#### 2.1.2. Exclusion Criteria

Exclusion criteria were psychomotor retardation that impaired the ability to provide informed consent, severe systemic illnesses (congenital or acquired syndromes and unstable metabolic disorders), any form of immunodeficiency (congenital, acquired, or medication-induced deficiencies), a current or past malignancy, chromosomal abnormalities, severe coagulopathy, and bone or ossification disorders. Individuals with a history of alcohol, narcotics, or medication abuse, heavy nicotine use (over 20 cigarettes/day), or the use of cortisone, bisphosphonates, or denosumab derivatives were also excluded. Non-compliance was another disqualifying factor.

### 2.2. Randomization

Participants were assigned to three parallel groups differing in their antibiotic regime based on the intraoperative extraoral storage time (EST) of the transplant: Group 1 had an EST of 0–3 min, Group 2 had an EST of 4–6 min, and Group 3 had an EST of 7–15 min, respectively. Random assignment was not approved by the ethics committee due to the young age of the participants and the adherence to Good Clinical Practice guidelines. Regarding EST, scientific evidence suggests its importance for maintaining the integrity of the tooth and periodontal apparatus [26,27], which, in turn, has a significant impact on the clinical outcome. This suggests that EST is the relevant distinguishing feature for group classification in this study. Patient enrollment, group assignment, and surgical procedures were all performed by a dentist with nearly 30 years of maxillofacial surgery experience. Allocation to groups took place during the surgery. When patients underwent multiple canine transplants, the tooth selected for study monitoring was chosen randomly by an independent assistant who drew lots from a concealed box.

### 2.3. Interventions and Follow-Ups

After obtaining a detailed medical and dental history and informed written consent, autogenous tooth transplantation was scheduled. To reduce the need for multiple surgeries, additional procedures like wisdom tooth removal were also performed during the transplantation. All operations were performed under general anesthesia using Propofol and transnasal endotracheal intubation. After recovery, patients were discharged. The procedure included creating a mucoperiosteal flap via a marginal incision, preparing a new socket with osteotomy, carefully extracting the tooth without damaging the root, and transplanting it. The tooth was temporarily stored in a solution of dexamethasone, doxycycline, and saline until reinsertion [28,29]. The transplant was secured with a saliva-tight adhesive fixation, and the surrounding soft tissue was closed with interrupted sutures. Postoperative follow-ups occurred on days 1, 7, and 21 for wound inspection, suture removal, and splint removal, respectively. For patients undergoing orthodontic treatment, fine-tuning, such as bracketing and moving the transplant, began in the fourth postoperative week. If orthodontic treatment was not needed or had already been completed, the transplant could remain unsplinted from the fourth week if clinically stable. All participants were prescribed ibuprofen (200–600 mg) as needed for pain. Antibiotic therapy varied by group: Group 1 received no antibiotics; Group 2 received a single intraoperative 100 mg i.v. dose of doxycycline; and Group 3 received the same intraoperative i.v. dose followed by a postoperative oral dose of 50–100 mg doxycycline (based on body weight) twice daily for five days. In case of postoperative infection, treatment involved early suture removal, saline irrigation, and, if needed, oral Amoxicillin/Clavulanic acid (500/125 mg or 875/125 mg, based on body weight) twice daily for five days. All follow-ups were conducted by the same dentist.

### 2.4. Outcomes

Two outcomes were defined as dependent variables: pain intensity, recorded via patient questionnaires, and clinically diagnosed signs of pathology, with cumulative scores calculated for both. The sum score of pain intensity (SPI) was based on the numerical rating scale (NRS) from 0 (no pain) to 10 (worst imaginable pain) at postoperative days 0, 1, 3, 7, 10, 15, and 21, resulting in a potential SPI range from 0 to 70. Similarly, the sum score of signs of pathology (SSP) was calculated by noting the presence (1) or absence (0) of various clinical signs—redness, swelling, pain, warmth, secretion, bleeding, fibrin coating, wound dehiscence, loss of protective blood clots, and reduced general condition—at days 7 and 21—with an SSP range of 0 to 20. This method aimed to track outcomes over the entire observation period. SSP was determined as the primary outcome because it is considered more specific to the transplantation situation and can be assessed more objectively due to documentation by an independent rater. In contrast, SPI was defined as the secondary outcome, as it represents a more general parameter that is inherently more subjective, being self-reported by the patients.

Independent or influencing variables were defined as follows: antibiotic regimen groups, sex, age, nicotine abuse, condition of the tooth apex (open or closed), preoperative ankylosis of the transplant, severity of displacement (mild, moderate, or severe), the jaw where the transplantation was performed (maxilla or mandible), the total number of simultaneous canine transplants, the total number of simultaneous surgical interventions, orthodontic extrusion of the transplant before autotransplantation, extraoral storage time (EST) of the transplant (to ensure comparability within a group), and the occurrence of intraoperative complications. Preoperative ankylosis was determined by radiological evidence (partially dissolved periodontal ligament or initial replacement resorption) and/or clinical findings (hyper-resonant or percussive sound) [30]. Similarly, the parameter “severity of displacement” was evaluated, considering the degree of deviation from the orthograde eruption path and/or the difficulty of surgical retrieval [31]. An overview of independent and dependent variables is given in Table 1.

The data collected were recorded in Microsoft Excel (Version 16.16.27, Microsoft Corporation, Redmond, WA, USA), analyzed, and graphically depicted using RStudio Open Source (Version 2024.09.1, Posit PBC, Boston, MA, USA). Descriptive statistics were calculated (mean, standard deviation (SD), range, median, and percentage), and normal distribution was checked using the Shapiro–Wilk test. To evaluate the correlation between independent variables and dependent variables (outcome parameters), univariate linear regression was employed with an alpha level set at 5% [32]. The effect size was measured using partial eta-squared statistics (ηP^2^), allowing for the estimation of the strength of influence of significant independent variables. A value of 0.01 indicated a small effect, 0.06 a medium effect, and 0.14 a large effect [33].

## 3. Results

### 3.1. Patient Data

**Figure 1:**
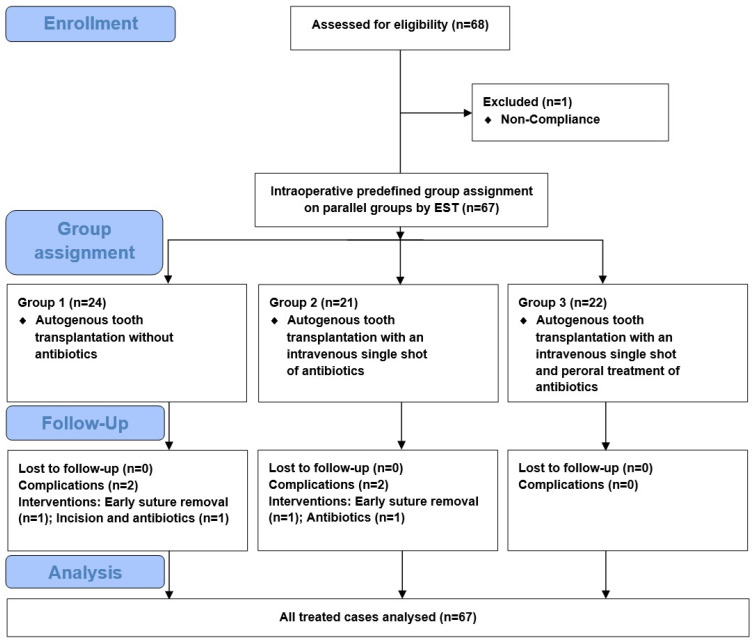
Flow of participants.

As depicted in the flow chart (Figure 1), 67 patients were enrolled in the study (43 females, 64%; 24 males, 36%) with ages ranging from 11 to 37 years (mean of 15.6 years, median of 14 years). Group 1 included 24 patients (EST 0–3 min), receiving no antibiotics; Group 2 involved 21 patients (EST 4–6 min), receiving intraoperative i.v. single-shot antibiotics; and Group 3 consisted of 22 patients (EST 7–15 min) receiving intraoperative i.v. and 5-day postoperative antibiotics. Among the cohort, six patients (9%) reported nicotine abuse (10–20 cigarettes per day). Anamnesis revealed that 17 patients (25%) had pre-existing conditions, i.e., grass/pollen allergy (9 patients), penicillin allergy (3 patients), hypothyroidism (2 patients: one post-thyroidectomy and one congenital thyroid aplasia), and ADHD (2 patients). Medication usage was noted in 11 patients (16%), involving antihistamines (5 patients), methylphenidate (2 patients), and L-thyroxine (2 patients).

### 3.2. Operation Data

Data were not normally distributed (Shapiro–Wilk test; *p* < 0.05). An overview of the assessed variables is given in Table 1. Of the 67 transplanted teeth, 19 (28%) had open apices, while 48 (72%) had closed apices. Preoperative ankylosis was present in 15 (22%) teeth. The severity of tooth displacement was classified as mild in 14 (21%) cases, moderate in 21 (31%) cases, and severe in 32 (48%) cases. The distribution of the transplanted canines across the dental quadrants was as follows: tooth 13 (n = 30, 45%), tooth 23 (n = 21, 31%), tooth 33 (n = 12, 18%), and tooth 43 (n = 4, 6%). Most transplants were placed in the maxilla (n = 51, 76%), while 16 (24%) were placed in the mandible.

Fourteen patients underwent more than one canine transplant, with two patients receiving three transplants and twelve patients receiving two. Additional procedures included wisdom tooth removal with follicular soft tissue transplantation to the canine site, exposure and bracketing of another canine, premolar/molar transplantation, and dental implantation. Surgical interventions involved one region in 40 patients, while the remaining patients had interventions in two to six regions.

The average EST of the transplants was 4.99 ± 2.98 min (mean ± SD; range 1–12 min). The majority of patients (n = 62, 93%) were undergoing orthodontic therapy at the time of transplantation, with 16 patients (24%) experiencing unsuccessful preoperative orthodontic extrusion of the transplanted tooth for 3 to 36 months (mean duration of 19.69 months).

Intraoperative complications occurred in 13 patients (19%), including transplant injuries (n = 4) and detected resorptions of the transplanted tooth (n = 3). Within the first week postoperation, complications arose in two patients (3%), necessitating additional antibiotics and premature suture removal. After two and three weeks, complications occurred in two more patients, including an irritating residual suture and an abscess, which were treated with suture removal and an incision with antibiotics, respectively. In contrast, medication side effects were reported by six patients (9%) during the first week, including diarrhea, nausea, constipation, vertigo, and arterial hypotension, but no side effects were reported after two and three weeks.

### 3.3. Primary and Secondary Outcome (SSP, SPI)

**Figure 2:**
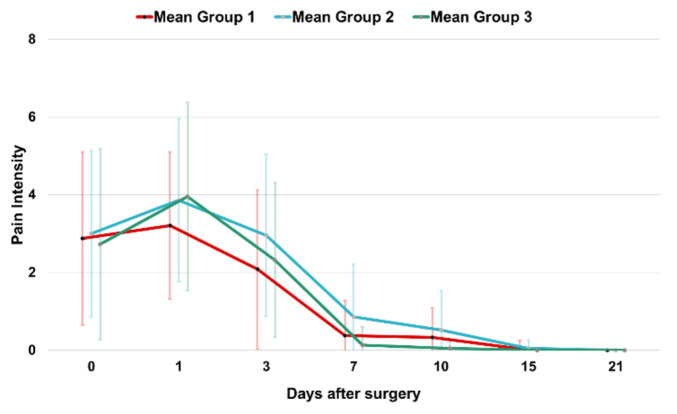
The course of pain intensity over the first three postoperative weeks. Mean values and standard deviations are given for each group.

Figure 2 illustrates the course of pain intensity over the first three postoperative weeks for Groups 1–3. Generally, pain peaked on the first postoperative day and had subsided almost completely by the time suture removal occurred after seven days. However, some patients achieved pain relief only between ten and twenty-one days after surgery.

**Figure 3:**
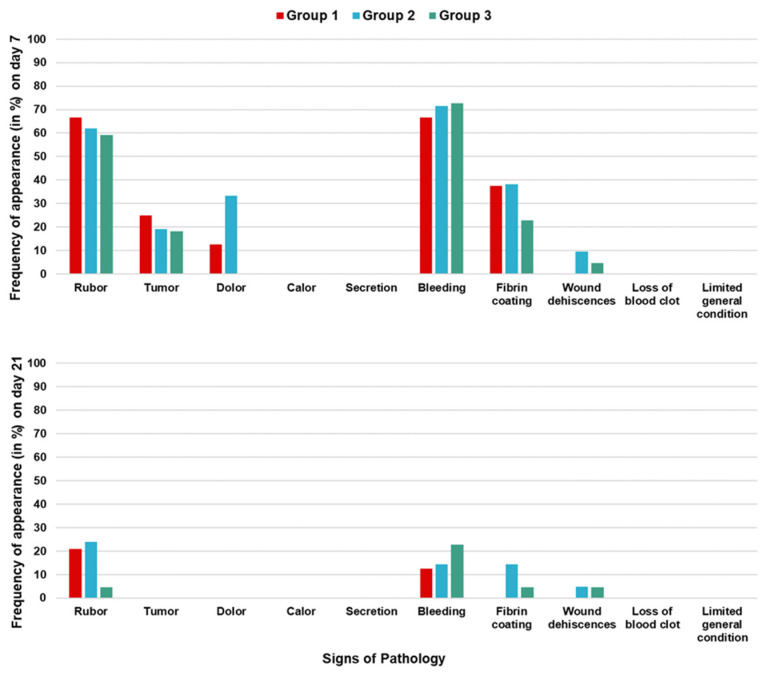
Frequency of signs of pathology on the 7th and the 21st postoperative days.

Regarding the signs of pathology, Figure 3 shows the frequency of occurrence in each group at day 7 and day 21 postoperatively. Redness, swelling, tenderness, bleeding, and fibrin deposits were commonly observed in the surgical area on day seven, but these signs had mostly regressed by the three-week mark.

**Figure 4:**
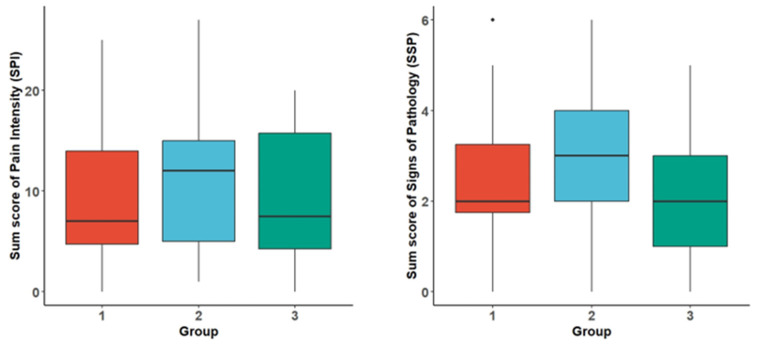
Sum score of pain intensity (SPI) and sum score of signs of pathology (SSP) over the first three postoperative weeks. The boxplots display the minimum/maximum, the median (horizontal line within the box), and the interquartile range for the respective groups.

Figure 4 presents the cumulative scores for pain intensity (SPIs) and signs of pathology (SSP) for the first three weeks, separated by the three groups. The data reveal no distinct differences in these scores among the groups.

### 3.4. Factors Influencing the SSP

No significant correlation regarding the sum score of signs of pathology (SSP) could be found for the assigned groups for sex, apex condition, severity of displacement, jaw location, the number of simultaneous canine transplants, orthodontic extrusion on the transplanted tooth, EST, or the occurrence of intraoperative complications (linear regression; *p* > 0.05). In contrast, age, nicotine abuse, and the sum of simultaneous interventions significantly increased the SSP (linear regression; *p* = 0.024, *p* = 0.029, *p* = 0.001). In detail, each additional year of age increased the SSP marginally by 0.1 points with a medium effect size (ηP^2^ = 0.08). Moreover, nicotine abuse showed a substantial negative influence, increasing the SSP by 1.5 points with a medium effect size (ηP^2^ = 0.07). Finally, an increase in SSP by 0.6 points for each simultaneously conducted intervention was observed, with the effect size being the highest in this context (ηP^2^ = 0.17).

Detailed information on the linear regression analysis between the significantly influencing parameters and SSP is given in Supplementary Figure S1A–C and Supplementary Tables S1–S3.

### 3.5. Factors Influencing the SPI

No significant influence on the sum score of pain intensity (SPI) could be found for the assigned groups for sex, age at surgery, nicotine abuse, apex condition, severity of dis-placement, jaw location, nor the number of simultaneous canine transplants, EST, or the occurrence of intraoperative complications (linear regression; *p* > 0.05). In contrast, significant correlations emerged with the influencing factors of preoperative ankylosis, the sum of interventions, and preoperative orthodontic extrusion attempts on the transplant (TX) (linear regression; *p* = 0.020, *p* = 0.002, and *p* = 0.035). Preoperative ankylosis increased the SPI by 4.5 points with a medium effect size (ηP^2^ = 0.08). Preoperative orthodontic extrusion of the transplant caused an increase in SPI by 4.0 points with a medium effect size of ηP² = 0.07. Each additional simultaneous intervention increased the SPI by 2.1 points, showing a strong effect size (ηP^2^ = 0.14).

Detailed information on the linear regression analysis between the significantly influencing parameters and SPI is given in Supplementary Figure S2A–C and Supplementary Tables S4–S6.

## 4. Discussion

The aim of this study was to investigate the potential influence of three different antibiotic regimens on the initial healing of autogenous canine transplants in conjunction with patient-specific variables. The current literature provides no clear recommendations for antibiotic therapy in dental transplantations [34,35]. For similar dental surgical procedures, major reviews either find no indication for routine systemic antibiotic use, as seen in the replantation of traumatically avulsed teeth [21] or report inconsistent findings, as in the case of oral implants [36]. Considering the potential side effects of antibiotics in a predominantly adolescent population [37] and the importance of minimizing unnecessary antibiotic use under the concept of antibiotic stewardship [38], it is crucial to review their application in autotransplantation. Antibiotic stewardship aims to maximize therapeutic benefits while minimizing resistance induction and adverse effects.

Due to the concerns raised by the ethics committee about depriving participants of adjunctive antibiotic therapy through randomized allocation, the antibiotic regimens were instead assigned intraoperatively based on EST (extraoral storage time), which is a significant prognostic factor in tooth replantation and transplantation. Importantly, the EST in all groups remained under the 60 min threshold (median times of 2, 4, and 8 min; Table 1) for a maximum of 12 min. This threshold has been established as a landmark for the survival of living cells within the periodontal apparatus, while outcomes improve further with EST under 20 min [26,27]. However, because antibiotic regimens could not be randomly allocated due to ethical restrictions, the assessment of the independent effects of both, antibiotics and EST on initial healing was limited. Nevertheless, the variety of patient ages and stages of tooth development, including cases with ankylosis, supports the generalizability of the findings to clinical practice.

Regarding signs of pathology and accumulated pain intensity, no significant correlation was found with EST group affiliation. This may indicate that the differences in EST between groups were too minor to negatively impact healing or that the antibiotic regimens applied did not significantly enhance healing outcomes. Further research is needed to clarify these findings.

Factors that showed a significant influence on the accumulated pathology signs included patient age, nicotine use, and the number of simultaneous surgical procedures. The association between advancing age and increased pathology signs is consistent with studies documenting a decline in the regenerative capacity of oral tissues with age [39]. Reduced blood flow and angiogenesis in adult patients may prolong wound healing, while increased bone mineralization in fully grown individuals may necessitate more invasive procedures [40,41]. Additionally, comorbidities and the use of medications that compromise wound healing are more common in adults [42], along with higher rates of nicotine abuse [43]. Nicotine’s detrimental effects on wound healing are well-established and result from peripheral vasoconstriction, reduced perfusion, and local inflammation induced by smoke exposure [44,45]. The association between multiple simultaneous surgical procedures and increased pathology signs may be due to swelling and pain limiting oral hygiene, leading to deposits and superficial inflammation at the wound site [46,47]. These findings suggest that optimizing the surgical plan and maintaining excellent postoperative care are critical to minimizing complications.

Factors that significantly influenced the accumulated pain intensity were also identified, including pre-existing ankylosis, prior unsuccessful orthodontic extrusion, and the number of simultaneous surgical interventions. The link between increased invasiveness (e.g., in the case of an increased number of interventions) and heightened swelling and pain is clinically evident, as greater tissue trauma is expected to result in more pronounced inflammatory responses [48]. Performing multiple procedures in a single session is well-documented in the literature as being time- and cost-efficient and reduces the need for multiple general anesthesia in adolescents, which could have potential long-term neurological effects [49,50]. Pre-existing ankylosis presents a significant challenge, as the transplantation of an ankylosed tooth typically requires extensive osteotomy to remove the tooth due to bone penetration and the loss of periodontal ligament integrity [51]. This complexity likely explains the increased pain intensity in such cases. Similarly, unsuccessful orthodontic extrusion often leads to scarring or chronic inflammation caused by prolonged exposure to foreign materials such as brackets and traction chains. Reactive ankylosis can also develop due to trauma from applied forces, particularly if the periodontium was damaged during the procedure [10].

In addition to the identified factors influencing initial healing, experience also shows that correct indication, good patient compliance, and the optimal timing of surgical measures—ideally before the definitive closure of the apical foramen but with sufficient root maturity—are important. The practitioner’s experience likewise plays a crucial role in ensuring a successful prognosis.

This study’s limitations include restricted sample size, a single-center design with one surgeon, and potential bias due to the antibiotic regimen being assigned based on EST, as described above. The first two factors may weaken generalization, as the findings could be more robust if a larger patient population was studied [52] and if multiple centers with surgeons of varying experience levels or differing surgical techniques were involved [53]. A further limitation may arise from multiple interventions (e.g., simultaneous removal of wisdom teeth), which were performed within the same surgical session as canine autotransplantation to avoid additional general anesthesia. This could have influenced the patients’ reported assessment of pain intensity at the site of the autotransplantation, as distinguishing between different surgical areas might be difficult for the patient.

## 5. Conclusions

The results suggest that personalized rather than routine antibiotic therapy might be appropriate for initial healing in canine tooth transplantation, which is in line with the principle of antibiotic stewardship. Deteriorating factors include prolonged EST, preoperative ankylosis, orthodontic extrusion, an increased number of surgical sites, age, and nicotine abuse.

## Figures and Tables

**Table 1:** Independent and dependent variables. The categories, possible range of values, and data are given for all groups.

Subtype	Variable	Range/Categories	All Groups	Group 1	Group 2	Group 3
**Independent**	Group	1, 2, 3	67 participants (100%)	24 participants (36%)	21 participants (31%)	22 participants (33%)
	Sex	female, male	43 females (64%) 24 males (36%)	12 females (50%) 12 males (50%)	14 females (67%) 7 males (33%)	17 females (77%) 5 males (23%)
	Age	11–99 years	median of 14 years (+/−4.9)	median of 12 years (+/−5.2)	median of 15 years (+/−5.6)	median of 14.5 years (+/−3.1)
	Nicotine abuse	no, yes	*n* = 6 with nicotine abuse (9%) *n* = 61 without nicotine abuse (91%)	*n* = 1 with nicotine abuse (4%) *n* = 23 without nicotine abuse (96%)	*n* = 3 with nicotine abuse (14%) *n* = 18 without nicotine abuse (86%)	*n* = 2 with nicotine abuse (9%) *n* = 20 without nicotine abuse (91%)
	Condition of tooth apex	open, closed	*n* = 19 with open apex (28%) *n* = 48 with closed apex (72%)	*n* = 13 with open apex (54%) *n* = 11 with closed apex (46%)	*n* = 3 with open apex (14%) *n* = 18 with closed apex (86%)	*n* = 3 with open apex (14%) *n* = 19 with closed apex (86%)
	Preoperative ankylosis	no, yes	*n* = 51 without ankylosis (76%) *n* = 16 with ankylosis (24%)	*n* = 18 without ankylosis (75%) *n* = 6 with ankylosis (25%)	*n* = 14 without ankylosis (67%) *n* = 7 with ankylosis (33%)	*n* = 19 without ankylosis (86%) *n* = 3 with ankylosis (14%)
	Severity of displacement	mild, moderate, severe	*n* = 14 with mild displacement (21%) *n* = 21 with moderate displacement (31%) *n* = 32 with severe displacement (48%)	*n* = 5 with mild displacement (21%) *n* = 8 with moderate displacement (33%) *n* = 11 with severe displacement (46%)	*n* = 4 with mild displacement (19%) *n* = 6 with moderate displacement (29%) *n* = 11 with severe displacement (52%)	*n* = 5 with mild displacement (23%) *n* = 7 with moderate displacement (32%) *n* = 10 with severe displacement (45%)
	Jaw	maxilla, mandible	*n* = 51 in the maxilla (76%) *n* = 16 in the mandible (24%)	*n* = 18 in the maxilla (75%) *n* = 6 in the mandible (25%)	*n* = 15 in the maxilla (71%) *n* = 6 in the mandible (29%)	*n* = 18 in the maxilla (82%) *n* = 4 in the mandible (18%)
	Total number of simultaneous canine transplantations	1–3	*n* = 53 with 1 transplantat (79%) *n* = 12 with 2 transplants (18%) *n* = 2 with 3 transplants (3%)	*n* = 18 with 1 transplantat (75%) *n* = 6 with 2 transplants (25%)	*n* = 16 with 1 transplantat (76%) *n* = 3 with 2 transplants (14%) *n* = 2 with 3 transplants (10%)	*n* = 19 with 1 transplantat (86%) *n* = 3 with 2 transplants (14%)
	Total number of simultaneous surgical interventions	1–6	*n* = 40 with 1 intervention (60%) *n* = 15 with 2 interventions (22%) *n* = 6 with 3 interventions (9%) *n* = 2 with 4 interventions (3%) *n* = 3 with 5 interventions (4%) *n* = 1 with 6 inteverntions (2%)	*n* = 14 with 1 intervention (58%) *n* = 9 with 2 interventions (38%) *n* = 1 with 4 interventions (4%)	*n* = 11 with 1 intervention (52%) *n* = 3 with 2 interventions (14%) *n* = 5 with 3 interventions (24%) *n* = 1 with 4 interventions (5%) *n* = 1 with 6 interventions (5%)	*n* = 15 with 1 intervention (68%) *n* = 3 with 2 interventions (14%) *n* = 1 with 3 interventions (4%) *n* = 3 with 5 interventions (14%)
	Preoperative orthodontic extrusion on the transplant	no, yes	*n* = 51 without extrusion (76%) *n* = 16 with extrusion (24%)	*n* = 19 without extrusion (79%) *n* = 5 with extrusion (21%)	*n* = 13 without extrusion (62%) *n* = 8 with extrusion (38%)	*n* = 19 without extrusion (86%) *n* = 3 with extrusion (14%)
	Extraoral storage time (EST)	0–15 min	median of 4 minutes (+/−3.0)	median of 2 minutes (+/−0.6)	median of 4 minutes (+/−0.7)	median of 8 minutes (+/−1.7)
	Intraoperative complication	no, yes	*n* = 54 without complication (81%) *n* = 13 with complication (19%)	*n* = 20 without complication (83%) *n* = 4 with complication (17%)	*n* = 14 without complication (67%) *n* = 7 with complication (33%)	*n* = 20 without complication (91%) *n* = 2 with complication (9%)
**Dependent**	Sum score of Pain Intensity (SPI)	0–70	median of 8 (+/−6.7)	median of 7 (+/−6.4)	median of 12 (+/−7.2)	median of 7.5 (+/−6.3)
	Sum score of Signs of Pathology (SSP)	0–20	median of 2 (+/−1.6)	median of 2 (+/−1.5)	median of 3 (+/−1.6)	median of 2 (+/−1.5)

## Data Availability

The data presented in this study are available on request from the corresponding author since the sharing of pseudonymized raw data with third parties was not foreseen in the ethics application approved by the ethics committee responsible for this study. Should there be collegial interest in the data, a request for data sharing should be submitted to the ethics committee responsible.

## Supplementary Materials

The following supporting information can be downloaded at https://www.mdpi.com/article/10.3390/jcm14030821/s1. Figure S1: A–C: Linear relationships between the outcome parameter of the sum score of signs of pathology (SSP) and the influencing factors of (A) age, (B) nicotine abuse, and (C) sum of interventions; Figure S2: A–C: Linear relationships between the outcome parameter of the sum score of pain intensity (SPI) and the influencing factors of (A) preoperative ankylosis, (B) sum of interventions, and (C) orthodontic extrusion on the transplant (TX); Table S1: Coefficients of linear regression between the influencing parameter of age at surgery and the outcome parameter of SSP; Table S2: Coefficients of linear regression between the influencing parameter of nicotine abuse and the outcome parameter of SSP; Table S3: Coefficients of linear regression between the influencing parameter of the sum of interventions and the outcome parameter of SSP; Table S4: Coefficients of linear regression between the influencing parameter of preoperative ankylosis and the outcome parameter of SPI; Table S5: Coefficients of linear regression between the influencing parameter of the sum of interventions and the outcome parameter of SPI; Table S6: Coefficients of linear regression between the influencing parameter of orthodontic extrusion on the TX and the outcome parameter of SPI.
