# Supplementary material for: Autogenous Tooth Transplantation of Canines—A Prospective Clinical Study on the Influence of Adjunctive Antibiosis and Patient-Related Risk Factors During Initial Healing
Source: J Clin Med. 2025 Jan 26;14(3):821. doi: 10.3390/jcm14030821 (PMC11818325; doi:10.3390/jcm14030821)
Supplement: Supplementary file 1 [file jcm-14-00821-s001.zip › Supplementary Figures.pdf]

Supplementary Figures

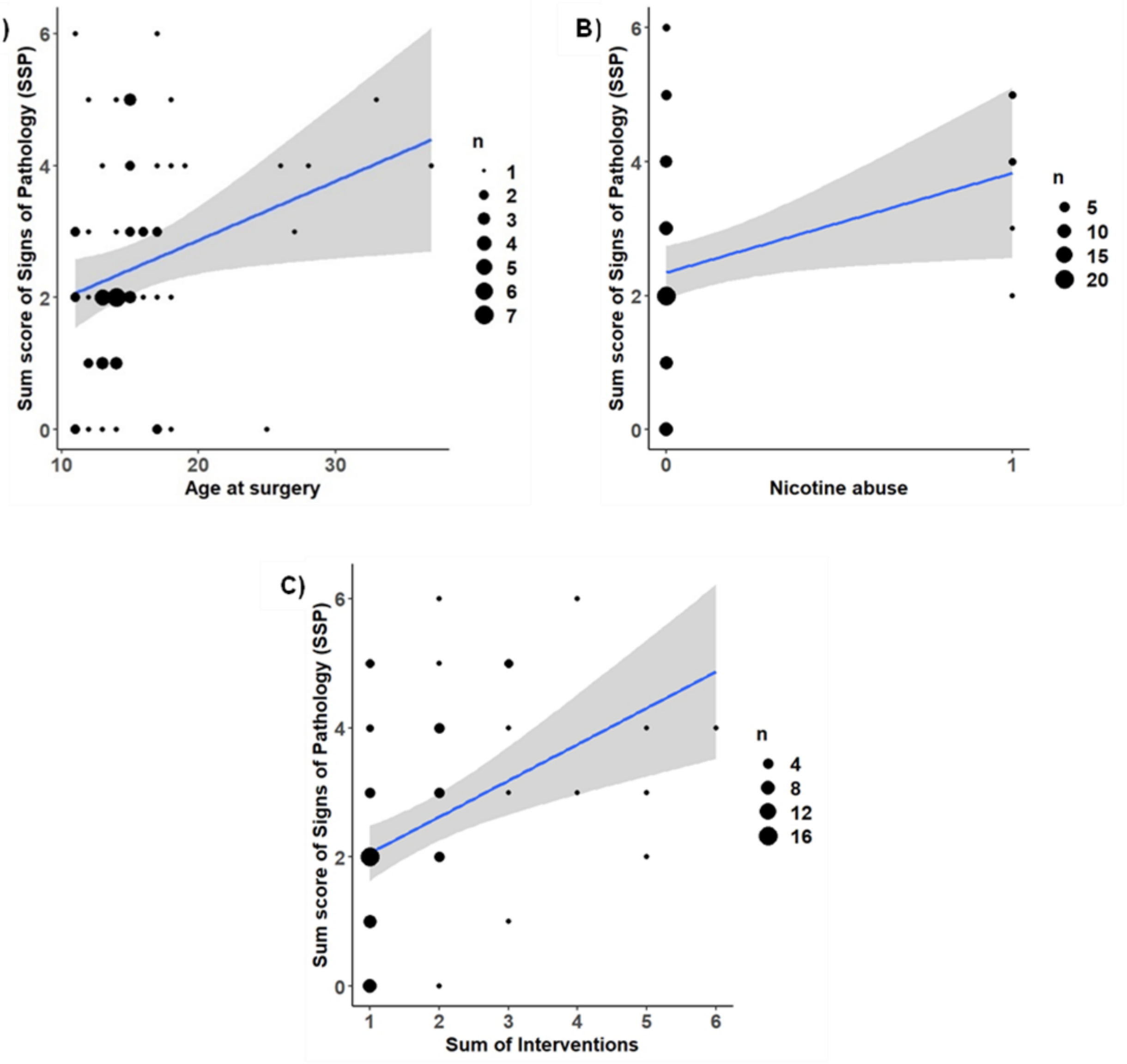

Figure S1. A-C Linear relationships between the outcome parameter of the sum score of signs of pathology (SSP) and the influencing factors of A) age, B) nicotine abuse, and C) sum of interventions.

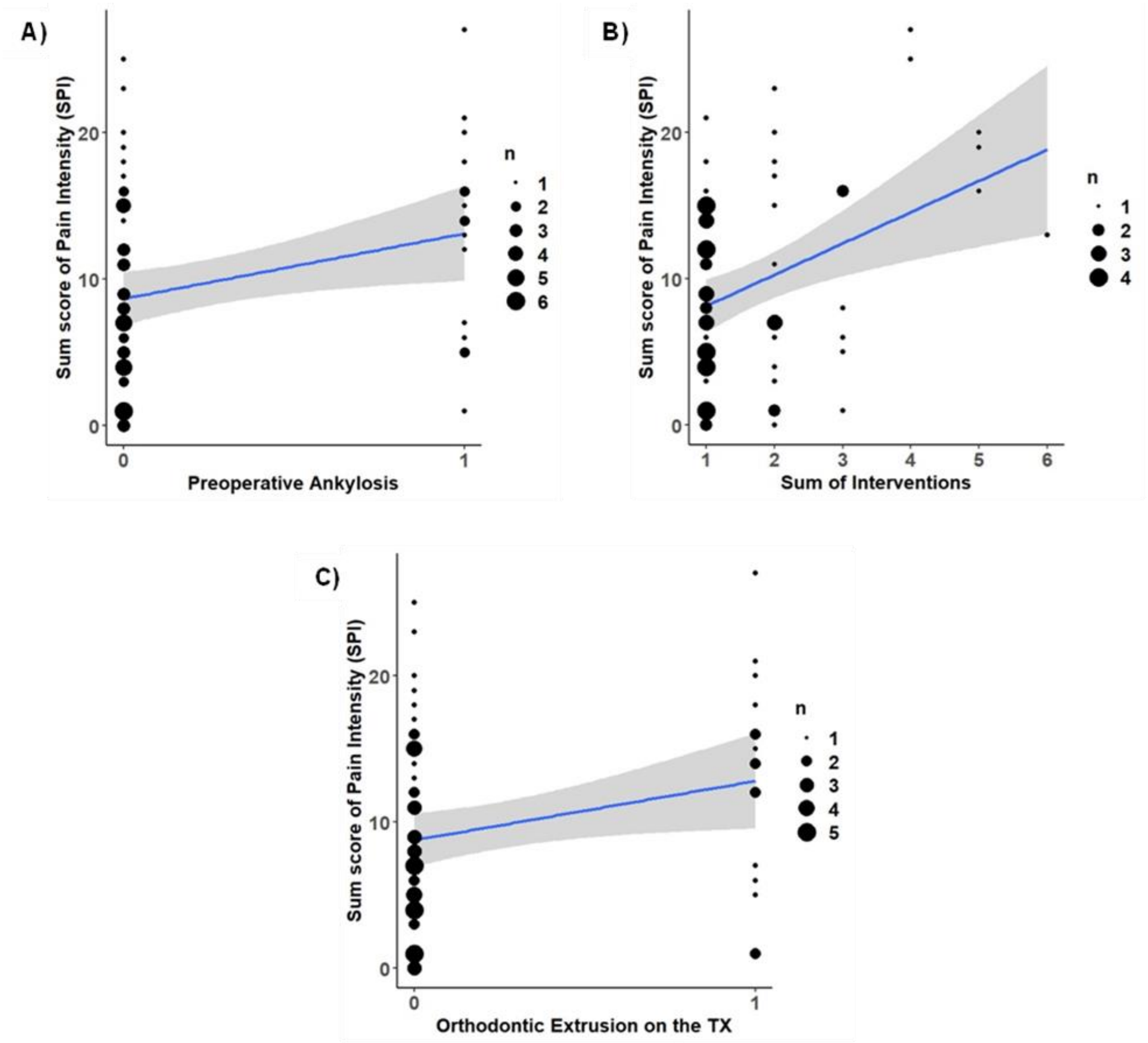

Figure S2. A-C Linear relationships between the outcome parameter of the sum score of pain intensity (SPI) and the influencing factors of A) preoperative ankylosis, B) sum of interventions, and C) orthodontic extrusion on the transplant (TX).
