# Supplementary material for: Autogenous Tooth Transplantation of Canines—A Prospective Clinical Study on the Influence of Adjunctive Antibiosis and Patient-Related Risk Factors During Initial Healing
Source: J Clin Med. 2025 Jan 26;14(3):821. doi: 10.3390/jcm14030821 (PMC11818325; doi:10.3390/jcm14030821)
Supplement: Supplementary file 1 [file jcm-14-00821-s001.zip › Supplementary Tables.pdf]

Supplementary Tables

Table S1. Coefficients of linear regression between the influencing parameter of age at surgery and the outcome parameter of SSP.

Coefficients:	Estimate	St. Error	t-value	p-value		Eta2	95% CI
(Intercept)	1.08269	0.63297	1.710	0.0919		-	-
Age at surgery	0.08944	0.03870	2.311	**0.0240**	*	**0.08**	[0.01, 1.00]

Table S2. Coefficients of linear regression between the influencing parameter of nicotine abuse and the outcome parameter of SSP.

Coefficients:	Estimate	St. Error	t-value	p-value		Eta2	95% CI
(Intercept)	2.3443	0.2000	11.721	<2e-16	***	-	-
Nicotine abuse	1.4891	0.6683	2.228	**0.0293**	*	**0.07**	[0.00, 1.00]

Table S3. Coefficients of linear regression between the influencing parameter of the sum of interventions and the outcome parameter of SSP.

Coefficients:	Estimate	St. Error	t-value	p-value		Eta2	95% CI
(Intercept)	1.4957	0.3226	4.637	1.76e-05	***	-	-
Sum of Interv.	0.5623	0.1532	3.670	**0.000491**	***	**0.17**	[0.05, 1.00]

Table S4. Coefficients of linear regression between the influencing parameter of preoperative ankylosis and the outcome parameter of SPI.

Coefficients:	Estimate	St. Error	t-value	p-value		Eta2	95% CI
(Intercept)	8.6667	0.9103	9.520	6.06e-14	***	-	-
Preop. Ankylosis	4.4583	1.8628	2.393	**0.0196**	*	**0.08**	[0.01, 1.00]

Table S5. Coefficients of linear regression between the influencing parameter of the sum of interventions and the outcome parameter of SPI.

Coefficients:

	Estimate	St. Error	t-value	p-value		Eta2	95% CI
(Intercept)	5.9928	1.3736	4.363	4.69e-05	***	-	-
Sum of Interv.	2.1409	0.6524	3.282	**0.00166**	**	**0.14**	[0.04, 1.00]

Table S6. Coefficients of linear regression between the influencing parameter of orthodontic extrusion on the TX and the outcome parameter of SPI.

Coefficients:

	Estimate	St. Error	t-value	p-value		Eta2	95% CI
(Intercept)	8.7647	0.9173	9.554	5.29e-14	***	-	-
Orthod. Extrusion	4.0478	1.8772	2.156	**0.0348**	*	**0.07**	[0.00, 1.00]
